# Non–high-density lipoprotein fractions are strongly associated with the presence of metabolic syndrome independent of obesity and diabetes: a population-based study among Iranian adults

**DOI:** 10.1186/s40200-017-0306-6

**Published:** 2017-06-07

**Authors:** Saeed Ghodsi, Alipasha Meysamie, Mehrshad Abbasi, Reza Ghalehtaki, Alireza Esteghamati, Masoud M. Malekzadeh, Fereshteh Asgari, Mohammad M. Gouya

**Affiliations:** 1 Department of Community and Preventive Medicine, School of Medicine, Tehran University of Medical Sciences, Tehran, Iran; 2 Endocrinology and Metabolism Research Center (EMRC), Vali-Asr Hospital, School of Medicine, Tehran University of Medical Sciences, Tehran, Iran; 3 Radiation Oncology Research Center, Cancer Institute, Tehran University of Medical Sciences, Tehran, Iran; 4 Digestive Disease Research Center, Shariati Hospital, Tehran University of Medical Sciences, Tehran, Iran; 5 Center for Disease Control, Ministry of Health and Medical Education, Tehran, Iran

**Keywords:** Non-HDL cholesterol, Metabolic syndrome, Cut-points, Diabetes mellitus, SuRFNCD-2007

## Abstract

**Background:**

Non-HDL-C as a valuable predictor of premature atherosclerosis, coronary events like first Myocardial infarction and cardiovascular mortality has a high accuracy of measurement both in fasting and non-fasting individuals. Metabolic syndrome (MetS) can promote the development of diabetes mellitus, endothelial dysfunction and atherosclerosis. A common pathway for cross linking of metabolic abnormalities and non-HDL-C has been suggested. In this study we aimed to describe the potential association between non-HDL cholesterol fractions and metabolic syndrome.

**Methods:**

Data of third national surveillance of the risk factors of non-communicable diseases (SuRFNCD-2007) were analyzed. We defined metabolic syndrome (MetS) according to the Adult Treatment Panel III (ATPIII) and International Diabetes Federation (IDF) criteria for 2125 subjects aging 25–64 years. The receiver operating characteristic (ROC) curves were used to determine the optimal cut-points for the diagnosis of MetS. The curves were depicted for non–high-density lipoprotein cholesterol (non-HDL-C) and difference of total non-HDL-C and LDL-C (Differential cholesterol or Diff-C) as predictors of MetS. Logistic regression was also performed in a complex sample analysis scheme.

**Results:**

The area under the curve (AUC) with 95% Confidence intervals of total non-HDL-C was computed. Values were 0.693 (0.670-0.715) for IDF-defined MetS and 0.719 (0.697-0.740) for ATPIII criteria. The optimal non-HDL-C cut-point we recommend for both criteria is 153.50 mg/dl (sensitivity: 75.7%, specificity: 57.2%, with ATPIII; sensitivity: 73.2%, specificity: 57.1%, with IDF). Using IDF criteria, the accuracy of predictors were greater in non-diabetic subjects. AUC of Diff-C in DM (−) vs. DM (+) were 0.786 (0.765-0.807) vs. 0.627(0.549-0.705). Adults with high non–HDL-C were 4.42 times more likely to have ATPIII-defined MetS (≥190 vs. < 190 mg/dL). Elevated Diff-C corresponded to increased risk of the MetS (ORs: 10.71 and 26.29 for IDF and ATP III criteria, respectively. All *P*-values <0.001).

**Conclusions:**

A significant robust association exists between non-HDL-C and MetS whether applying conventional or new thresholds.

## Background

Non–high-density lipoprotein cholesterol (Non-HDL-C) is known as a valuable predictor of premature atherosclerosis, coronary events like first Myocardial infarction and cardiovascular mortality. It is measured by subtracting the HDL component from total cholesterol concentration. In other words, non-HDL-C consists of atherogenic remnants including lipoprotein (a), VLDL (very-low-density lipoprotein), LDL, and IDL (intermediate-density lipoprotein). There is an increasing trend of evidences which support the role of Non-HDL-C in cardiovascular risk assessments [1–4]. It has been suggested that Non-HDL-C levels may reflect the risk of coronary heart disease (CHD) better than low-density lipoprotein Cholesterol (LDL-C) alone. In addition, a high accuracy of measurement in fasting and non-fasting individuals is available for non-HDL fraction. Indeed, a large number of adults with high non-HDL-C have normal to borderline levels of LDL-C. Nonetheless, many of them have CHD risk equivalents such as diabetes mellitus and metabolic syndrome [5–7].

Metabolic syndrome (MetS) is a constellation of risk factors characterized by central obesity, dyslipidemia, hypertension and hyperglycemia associated with insulin resistance. Presence of this profile can promote the development of diabetes mellitus, endothelial dysfunction and atherosclerosis [8, 9]. Such pathways, eventually lead to subsequent cardiovascular diseases especially CHD [10]. Pathophysiology of atherosclerosis seems to be a common pathway for cross linking of metabolic abnormalities and non-HDL-C. Furthermore, we are faced with a high prevalence of metabolic syndrome and a growing proportion of its components in Iran [11]. Hence, implication of non-HDL-C in clinical practice provides an optimal index in order to discriminate subjects with and without MetS. Thus we aimed to evaluate the association between non-HDL cholesterol and metabolic syndrome among Iranian adults. It has been established that a significant difference (>30 mg/dl) in non-HDL cholesterol and LDL concentrations contributes to elevated cardiovascular disease mortality [5]. Therefore, we also depicted the distributions of this lipoprotein particle (so called Diff cholesterol or Diff-C = [total non-HDL-C] **-** [LDL-C]) in study participants. The optimal cut-off points for the diagnosis of MetS were also determined using both of the surrogate measures among diabetic and nondiabetic subjects. These thresholds may improve the risk stratification of patients, in addition to better screening of subjects at increased risk of MetS with a simple instrument.

## Methods

### Study population

Third national survey around the risk factors of non-communicable diseases was launched in 2007 in all provinces of Iran; a multi-centric study with stratified clustered sampling design. To be representative of Iranian population, appropriate proportions had been considered including area of residence (urban–rural), age strata, Gender and all racial-ethnic groups. A total number of 5287 non-institutionalized participants aged 15–64 years had been enrolled in the survey. As explained in our previous reports, structured questionnaires based on WHO STEPS guidelines were used in SURFNCD-2007.The questionnaires were interview-administered; consisted of 6 major domains: Demographic features, physical activity, tobacco use, past medical history of diabetes and hypertension [12, 13]. Trained health care professionals collaborating with 40 medical schools (across the country) conducted the interviews and physical examinations. Anthropometric parameters and Blood pressure were determined using the standard protocol of the survey. Body mass index (BMI) was calculated as weight (in kilograms) divided by height (in meters) squared. Measurement of the covariates and further details about the survey have been extensively described elsewhere [12]. SURFNCD-2007 obtained the ethical approval of the Center for Disease Control (CDC) of Iran. Informed consent was obtained for data collection and blood sampling, separately.

In the present study, we have limited the analyses to participants older than 25 years due to different clinical definitions such as metabolic syndrome, hypertension, obesity, and dyslipidemia in younger subjects. Furthermore, frequency of cardiovascular disease (CVD) risk factors and their clinical impact is different in 15–25 year age group. Pregnant women and participants with missing data (for lipid profile and plasma glucose) were also excluded. Apparently healthy subjects who had not used lipid lowering medications were targeted. At last, a nested sample comprised of 957 men and 1168 women who met the above criteria was selected.

### Laboratory evaluations

Venous blood samples (10 ml) were drawn after an overnight fasting for a 12 h period. Specimens underwent centrifugation immediately. Principles of cold chain preservation was used to transfer the frozen samples (at < −70C^0^) to the central laboratory of Ministry of Health (Tehran, Iran). Fasting plasma glucose (FPG) was carried out by the enzymatic colorimetric method using glucose oxidize test (intra- and inter-assay coefficients of variation (CV) were 2.1% and 2.6%, respectively). Total cholesterol (TC), high density lipoprotein cholesterol (HDL-C), low density lipoprotein cholesterol (LDL-C) and triglycerides (TG) were measured by enzyme-linked assays on a multiple sample analyzer (Parsazmun, Karaj, Iran) [12]. Concentration of LDL-C was measured directly if TG levels exceeded 4.5 mmol/L. Otherwise, we computed the LDL-C concentration according to Friedwald formula [14].

Non–HDL-C concentration was calculated by subtracting HDL-C value from total cholesterol concentration. Diff-C was defined as the difference between total amount of non-HDL cholesterol and LDL-C (Diff-C = [non-HDL-C]-[LDL-C]). High sensitivity C-reactive protein (hs-CRP) was measured using a quantitative CRP kit (Parsazmun, Karaj, Iran) with an intra-assay CV of about 2.6%. Measurement of plasma Insulin was performed by radioimmunoassay (Immunotech, Prague, Czech Republic). Sensitivity was 0.5 μU/mL, and the upper limits of intra- and inter-assay coefficients of variation were 4.3 and 3.4, respectively [12].

### Definitions

Conventional recommended thresholds for non-HDL-C and Diff-C were used in our analysis. With regard to the risk of CVD, three levels of non-HDL-C have been offered to initiate life-style modification (followed by use of medications). These cut-points are 130, 160 and 190 mg/dl for non-HDL-C while Diff-C levels above 30 refer to an increased risk of CHD [7]. Diabetes mellitus (DM) was diagnosed by one of the followings: self-report of disease accompanied by use of prescribed medications or Fasting plasma glucose ≥126 mg/dl [15]. Homeostasis model assessment of insulin resistance (HOMA-IR) was computed by the following equation: {FPG (mg/dL) × Fasting Insulin (mU/mL)}/405 [16]. Total physical activity (TPA) was estimated by summation of scores for all kinds of activity with moderate or vigorous intensity (in METS × minutes). MET (Metabolic Equivalent of Task) is the ratio of a person’s working metabolic rate to the resting rate [17]. Hypertension was defined as systolic blood pressure ≥ 140 mmHg and/or diastolic blood pressure ≥ 90 mmHg, or current history of using anti-hypertensive drugs [18]. We identified participants with metabolic syndrome by the ATP-III and IDF criteria [19]. The ATP-III guideline, defines MetS when 3 or more of the following components are present:

1. Abdominal obesity (waist circumference ≥ 102 cm [men] or ≥88 cm [women]).
2. Triglyceride ≥150 mg/dL.
3. HDL-cholesterol <40 mg/dL (men) or <50 mg/dL (women).
4. Blood pressure ≥ 130/85 mmHg.
5. Fasting plasma glucose ≥100 mg/dL (or known diabetes).

According to IDF criteria, diagnosis of MetS was established for those with central obesity accompanied by any 2 of the following conditions: high plasma TG, high Blood pressure, hyperglycemia and low HDL-C as defined above (in ATPIII). Since IDF suggests national/regional cut-offs to define central obesity, we used the specific cut-points for the Iranian population [waist circumference (WC) > 90 cm for both genders] [11].

### Statistical analysis

Complex sample analysis method was performed using SPSS v.20 for Windows (Chicago, IL, USA). Data were weighted for age (10-year intervals), sex, and residence area (rural/urban) according to the results of the national census of Iran in 2006. The analysis plan was based on the original clusters of the survey, strata and calculated weights. Determined strata were consisted of 4 layers: the province, area of residence (urban/rural), age-groups and gender.

The receiver operating characteristic (ROC) curves were employed to determine the optimal cut-points (along with corresponding sensitivities and specificities) for the diagnosis of metabolic syndrome. Cut-off values pertaining to both non-HDL-C and Diff-C were compared between different definitions of MetS and diabetic versus nondiabetic subjects. Accuracy was described by area under the curve (AUC) with 95% Confidence Intervals. We determined the optimal thresholds using two common methods: the maximum Youden’s J index and the shortest distance (from the point (0, 1)).The former is defined as: [sensitivity- (1-specificity)] and the latter is the nearest value to the top left corner of the ROC curve ([(1 - sensitivity) ^2^ + (1 - specificity) ^2^]). An appropriate point is recognized where a plateau is found. In other words, such a point maximizes the Youden’s J index while minimizing the Distance index. We have also calculated the positive likelihood ratios (PLR) to have a conclusive concept for ruling-in the metabolic syndrome (as the end point) [20]. Comparison of the ROC results (AUC) was performed using the MedCalc software (v.15.8, USA).

Multivariate logistic regression analysis controlling for potential confounders was also performed. Stepwise adjustments resulted in 6 different models to examine the power of high non-HDL-C to discriminate cases with and without MetS (ORs, 95% CI). Traditional thresholds of non-HDL-C and quartiles of the sample were applied to achieve this goal. Probable confounding relationships were evaluated by a Chi-square test of homogeneity. These covariates were included in the logit models: age, sex, residence area, Hypertension, TPA, FPG, Insulin resistance (HOMA), smoking, Natural logarithm of C-reactive protein (Ln-CRP), and BMI.

We categorized Mets criteria into 6 groups according to number of positive criteria for each subject (0–5), then we assessed the trend of non-HDL-C and Diff-C values in these groups by ANOVA test. Continuous and categorical variables were expressed as mean ± SD (or SE) and percentages, respectively. CRP values were log-transformed (natural log scale) to reach a normal distribution. Statistical significance was considered as a *P*-value <0.05.

## Results

**Fig. 1:**
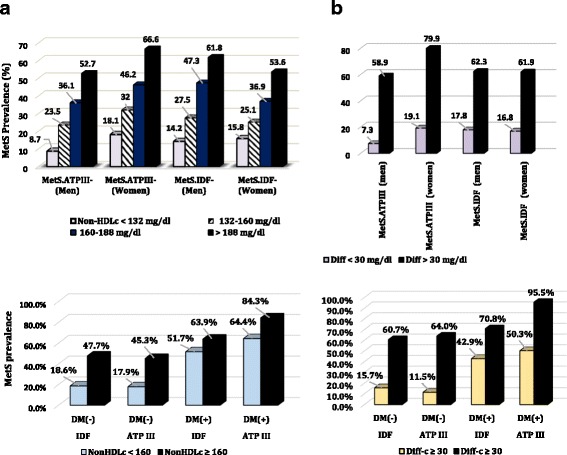
Prevalence of metabolic syndrome (ATP III and IDF) among different categories of Non-HDL-C fractions and both genders. Columns (**a**). and (**b**). refer to total (Non-HDL-C) and differential fractions (Diff-C: total Non-HDL – (LDL)) respectively. The inferior graphs show the compared prevalence of MetS in diabetics against nondiabetics

General clinical and biochemical characteristics of the study population by gender and DM status are presented in Table 1. The observed agreement between ATP III and IDF definitions (of metabolic syndrome) was considerable (kappa =0.711). Although the assessment in women and non-diabetic subjects revealed high levels of agreement, their counterparts showed weak to moderate concordance. The kappa statistic was 0.832 in women (against 0.553 in men). The prevalence of metabolic syndrome was strikingly higher in diabetic subjects (≥2 times for both criteria). However, application of different MetS criteria changed the prevalence among the two genders. Graphs in Fig. 1 represent the proportions of MetS among the subgroups of non-HDL cholesterol and Diff-C. MetS prevalence increased markedly by rising non-HDL-C levels (among the quartiles: Q1 < 132 mg/dl, 132 ≤ Q2 < 160, 160 ≤ Q2 < 188, Q4 ≥ 188). The *P*-value for trend was <0.0001. It was a similar pattern depicted for men and women (with both ATPIII and IDF criteria). By ATP III, women had greater proportions of MetS than men (when we compared the same quartiles of non-HDL-C; all *P*-values <0.0001). Nevertheless, using the IDF criteria determined higher prevalence of MetS in males (although the comparison was significant only for upper quartiles:Q3 and Q4). Metabolic syndrome was far more common in cases with high Diff-C compared with lower concentrations (Diff-C ≥ 30 versus <30 mg/dl; *P*-value <0.0001). Women had also greater proportions of ATPIII-defined MetS in both of the Diff-C subgroups (*P*-value <0.0001) while this predominance was not demonstrated for IDF-defined MetS (*P*-value: 0.322).

**Table 1:** General characteristics of the study participants

		Men (*n* = 957)	Women (*n* = 1168)	*P* -value	DM (−) *n* = 231	DM (+) *n* = 1894	*P* -value	Total (*n* = 2125)	Total NE ¶
Age (year)		39.2 ± 4.6 (38.9-39.5)	39.6 ± 4.2 (39.3-39.8)	**0.036**	38.7 ± 7.4 (38.3, 38.9)	47.0 ± 14.1 (45.2, 48.9)	**<0.001**	39.4 ± 4.5 (39.2 -39.6)	
BMI (kg/m2)		25.7 ± 4.6 (25.4- 26.0)	27.6 ± 5.5 (27.2,27.9)	**<0.001**	26.4 ± 5.6 (26.2, 26.7)	28.95 ± 7.58 (27.97, 29.93)	**<0.001**	26.6 ± 5.0 (26.4,26.9)	
Hypertension (%)		23.9% (19.8 -28.6)	29.6% (26.7 -32.7)	**0.003**	25.7% (23.8, 27.8)	55.3% (46.8, 63.5)	**<0.001**	26.8% (24.3 -29.5)	7.65
TG (mg/dl)		151.8 ± 90.2 (146.1, 157.5)	136.1 ± 74.9 (131.8140.4)	**<0.001**	139.4 ± 79.6 (135.9, 143.0)	190 ± 95.7 (177.6202.0)	**<0.001**	143.9 ± 30.5 (140.3, 147.5)	
HDL-C (mg/dl)		34.4 ± 8.3 (33.9, 35.0)	39.6 ± 11.7 (38.9,40.3)	**<0.001**	37.2 ± 11.0 (36.7, 37.7)	34.9 ± 10.8 (33.5, 36.3)	**0.003**	37.0 ± 10.0 (36.6,37.5)	
LDL-C (mg/dl)		127.9 ± 30.5 (126.0, 129.9)	132.8 ± 33.2 (130.9, 134.7)	**<0.001**	129.5 ± 35.0 (127.9, 131.1)	139.3 ± 55.7 (132.1, 146.5)	**<0.001**	130.4 ± 31.8 (129.0,131.7)	
TC (mg/dl)		192.8 ± 35.2 (190.6, 195.1)	199.6 ± 38.3 (197.4201.8)	**<0.001**	194.7 ± 41.9 (192.8, 196.6)	212.1 ± 59.0 (204.5, 219.8)	**<0.001**	196.3 ± 36.7 (194.7197.8)	
Non-HDL-C (mg/dl)		158.4 ± 46.2 (155.5, 161.3)	160.0 ± 42.2 (157.6, 162.4)	0.402	157.5 ± 41.8 (155.6, 159.4)	177.2 ± 63.0 (169.1, 185.3)	**<0.001**	159.2 ± 44.6 (157.3, 161.1)	
Diff-C (mg/dl)		30.5 ± 18.6 (29.3, 31.6)	27.2 ± 15 (26.4, 28.1)	**<0.001**	27.8 ± 16.3 (27.2, 28.7)	37.9 ± 18.9 (35.5, 40.4)	**<0.001**	28.8 ± 17.0 (28.1, 29.6)	
Ln CRP		1.5 ± 0.9 (1.4, 1.60)	1.5 ± 0.9 (1.4, 1.6)	0.736	1.5 ± 0.9 (1.4, 1.6)	1.5 ± 1.2 (1.3, 1.7)	0.882	1.5 ± 0.9 (1.4, 1.6)	
Diabetes Mellitus (%)		8.2% (6.3, 10.6)	9.4% (7.9, 11.3)	0.333			**<0.001**	8.8% (7.5, 10.3)	2.38
MetS (Prevalence %)	MetS ATPIII	29.2% (26.0, 32.7)	40.0% (37.1, 43.0)	**<0.001**	30.5% (28.3, 32.7)	77.7% (70.8, 83.3)	**<0.001**	34.7% (32.5, 36.9)	9.36
	MetS IDF. Ir	36.7% (33.2, 40.4)	32.2% (29.6, 35.0)	**0.030**	32.0% (29.7, 34.3)	59.8% (51.9, 67.3)	**<0.001**	34.5% (32.2, 36.7)	9.31
Current-Smokers (%)		18.4% (15.5, 21.6)	1.5% (0.9, 2.6)	**<0.001**	10.0% (8.4, 11.9)	8.4% (5.0, 13.7)	0.334	9.9% (8.4, 11.6)	2.67
Waist circumference (cm)		89.3 ± 15.7 (88.3, 90.3)	88.4 ± 14.4 (87.6,89.3)	0.975	88.2 ± 14.5 (87.5, 88.8)	96.0 ± 18.5 (93.6, 98.4)	**<0.001**	88.9 ± 15.2 (88.2, 89.5)	
HOMA		2.4 ± 3.6 (2.2, 2.7)	2.4 ± 2.0 (2.2, 2.5)	0.640	2.0 ± 1.5 (1.8, 2.1)	6.3 ± 9.2 (5.1, 7.5)	**<0.001**	2.4 ± 3.0 (2.3, 2.5)	
TPA (METS-minute)		5982.0 ± 316.4 (5361.2,6602.8)	2427.5 ± 3950.7 (2200.7,2654.4)	**<0.001**	4388.8 ± 8131.7 (4022.1, 4755.5)	2139.9 ± 3227.7 (1723.2, 2556.6)	**<0.001**	4190.5 ± 172.3 (3852.5,4528.5)	

Results have been expressed as mean ± SD (95% confidence interval)

¶ NE: National Estimates rounded to the nearest million according to computed weights. Bold *P* values indicate statistical significance

Participants with metabolic syndrome (by any criteria) had higher non-HDL-C and Diff-C concentrations rather than the remaining cases. For instance, the average (95% CI) of non-HDL-C was 179.11(176.04, 182.17) among MetS (+) subjects versus 148.67(146.50, 150.85) among the others if the ATP-III criteria was applied. Mean values (95% CI) of Diff-C were 39.54(38.35, 40.74) and 23.16(22.45, 23.88) for subjects with and without MetS (ATP III), respectively.

**Fig. 2:**
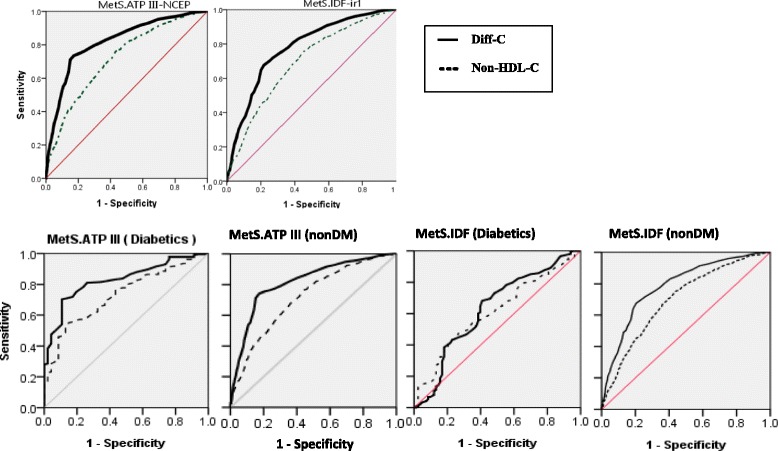
Age and sex standardized ROC curves of Non-HDL-C and Diff-C for diagnosis of metabolic syndrome. The diagnostic criteria for metabolic syndrome are ATP III (*left*) and IDF (*right*)

**Fig. 3:**
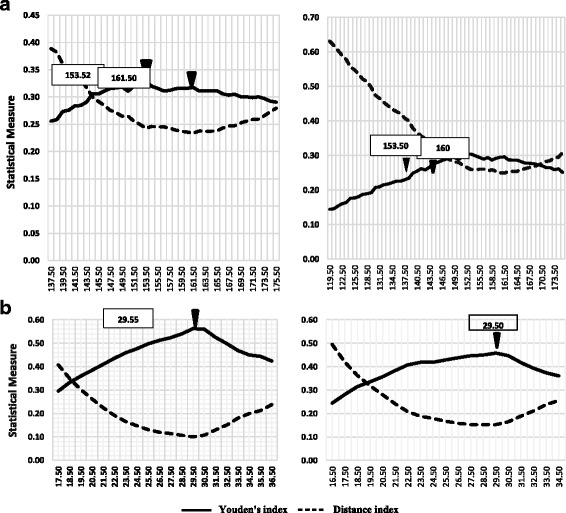
The optimal cut points of Non-HDL-C and Diff-C for diagnosis of metabolic syndrome. The diagnostic criteria for MetS are those recommended by the international diabetes federation (IDF) (*right*) and Adult Treatment Panel III (ATP III) (*left*). Panel **a** shows the distribution of Non-HDL-C and the panel **b** refers to Diff-C

Results of the ROC analyses were presented in Tables 2 and 3. Related ROC curves for all of the participants in addition to the graphs for subgroups of DM were also depicted in Fig. 2. Analyses were standardized for age and sex. Optimal cut-off points presented in the Table 2 have been determined based on the agreement of Youden’s J statistic and the Distance method. The overall distribution of both indexes for diagnosis of the metabolic syndrome are depicted in Fig. 3(by 2 panels for non-HDL-C and Diff-C). Since different levels of sensitivity and specificity might be used depending on the clinical situation, we have shown a variety of thresholds in Table 3.As an example, assume the non-HDL-C thresholds of 133 and 140 in order to diagnose ATPIII-defined MetS (pertaining to DM (+) and DM (−) status, respectively). Their corresponding pairs of sensitivity and specificity were:(88.8%, 35.7%) and (86.5%, 37%).Therefore the points are acceptable for screening of MetS. On the other hand, a non-HDL-C threshold of 169.5 (sen: 58.4%, spe: 73.9%) was also considered for diabetics which aids to reduce the false positives.

**Table 2:** Age and sex standardized ROC curve analysis of non–HDL-C and differential fraction (Diff-C) for MetS among Iranian adults aged 25–64 years, SURFNCD-2007

MetS definition		AUC	95% CI	*P*-value	Optimal cut-off (mg/dL)	Sensitivity (%)	Specificity (%)
A	ATP III	0.719	(0.697, 0.740)	<0.001	153.52	75.7	57.2
	ATP III in DM (−)	0.717	(0.693, 0.740)	<0.001	161.5	67.4	64.1
	​ATP III in DM (+)	0.733	(0.659, 0.807)	<0.001	175.5	55.1	84.8
	IDF	0.693	(0.670, 0.715)	<0.001	153.50	73.2	57.1
	​IDF in DM (−)	0.698	(0.674, 0.722)	<0.001	160	67	63.4
	​IDF in DM (+)	0.608	(0.534, 0.683)	<0.001	175.75	54	65.3
B	ATP III	0.819	(0.801, 0.838)	<0.001	29.55	73.3	82.9
	ATP III in DM (−)	0.817	(0.797, 0.834)	<0.001	30	72.4	88.3
	ATP III in DM (+)	0.828	(0.770, 0.887)	<0.001	30	70.3	89.1
	IDF	0.777	(0.757, 0.797)	<0.001	29.50	65.9	80.4
	​IDF in DM (−)	0.786	(0.765, 0.807)	<0.001	29.45	67.5	79.6
	​IDF in DM (+)	0.627	(0.549, 0.705)	<0.001	30	68.2	59

Panels A and B, refer to analysis and corresponding cut-points of Non-HDL-C and Diff-C fractions, respectively.

*P*-values test the null hypothesis of AUC = 0.5

**Table 3:** Various optional cut off points for the diagnosis of MetS with regard to similar sensitivities

MetS definition		Cut-point 1	Sen (%)	Spe (%)	Cut-point 2	Sen (%)	Spe (%)	Cut-point 3	Sen (%)	Spe (%)	Cut-point 4	Sen (%)	Spe (%)	Cut-point 5	Sen (%)	Spe (%)
A	ATP III	**133.5**	88.2	35.7	**146.5**	80.9	50.2	**155**	73.9	57.9	**164.5**	64.9	66.2	**170**	58	72.7
	ATP III in DM (−)	**133**	88.8	35.4	**146**	80.9	51.1	**153.5**	75.4	67.2	**165**	64.1	66.8	**170**	58.4	61.7
	ATP III in DM (+)	**140**	86.5	37	**149.5**	80	50	**154**	76.8	57.5	**164.5**	65.4	67.4	**169.5**	58.4	73.9
	IDF	**134.5**	86.7	35.4	**145**	80.5	48.1	**153**	75	55.5	**163.5**	64.5	64.2	169	58.	69.6
	​IDF in DM (−)	**133.5**	87.4	35.6	**145**	80.4	48.9	**153.5**	74.5	67.2	**164**	64	65.7	**169**	58	70.8
	​IDF in DM (+)	**150.2**	78	35.4	**154**	75	38.6	**157**	70	39.2	**162.5**	65.5	47	**169.5**	57.4	57.8
B	ATP III	**17.5**	93	36.5	**23.5**	82.8	62.1	**27.5**	76.1	76.2	**33**	61.7	86.4	**36.5**	52.3	90.1
	ATP III in DM (−)	**17.5**	93	36.9	**23.5**	82.7	63.4	**27.5**	76.4	76.1	**33**	60.8	86.6	**35**	54.7	88.6
	ATP III in DM (+)	**19.5**	89.7	37	**22.5**	83.8	52.2	**27.5**	75.1	80.4	**35**	61.6	89.1	**38**	54.9	89.1
	IDF	**17.5**	92.4	35.7	**21**	83.5	51.5	**25.5**	75.9	77	**31.5**	61	80.7	**34.5**	52	84.1
	​IDF in DM (−)	**17.5**	92.6	37.1	**21**	86.2	52	**26**	74.3	73.4	**31.5**	60.1	82.1	**33**	54.8	83.8
	​IDF in DM (+)	**23**	82.7	36.3	**25.5**	78.4	44.6	**27**	74.3	42.8	**34**	60.5	60.9	**36**	55.8	61.4

Panels A and B, refer to the cut-points of Non-HDL-C and Diff-C fractions (Bold numbers), respectively

Minimum value of specificity was considered 35% for all of the thresholds. A descending trend of sensitivities was depicted from the first to the fifth cut point

According to the computed AUC of the ROC curves in (Table 2), 3 comparative approaches were used:

### DM (+) vs. DM (−)

If the IDF criteria is set for metabolic syndrome, non-diabetic subjects had a greater area (AUC) than diabetics. Relative *P*-values were calculated: <0.0001 and 0.024 for Diff-C and non-HDL-C respectively. However, a significant difference was not identified applying the ATP III criteria. Corresponding *P*-values were: 0.718 for Diff-C and 0.681 for non-HDL-C.

### ATP III vs. IDF

The accuracy of lipoprotein fractions to identify metabolic syndrome was higher for ATP III definition rather than IDF regardless of DM status. We compared the following pairs of AUC: (0.819 vs. 0.777), (0.719 vs. 0.693), (0.828 vs. 0.627), (0.733 vs. 0.608), (0.817 vs. 0.786) and (0.717 vs. 0.698). *P*-values were determined: < 0.0001, 0.117, < 0.0001, 0.018, 0.049 and 0.296, respectively.

### Diff-C vs. non-HDL-C

We demonstrated that the Diff-C measure is able to predict MetS more accurately than total non-HDL-C. All *P*-values were <0.0001 except in 2 subgroups (of diabetic subjects). In other words, two comparisons were performed within diabetics: (AUC: 0.828 vs. 0.733) and (AUC: 0.627 vs. 0.608) for ATP III and IDF criteria, respectively. Corresponding *P*-values were: 0.041 and 0.720, respectively.

Subsidiary analyses of ROC curves were also performed. For instance, AUC with 95% CI was 55.8(52.2, 59.4) for Diff-C versus 55.4 (51.7, 59.2) for non-HDL cholesterol. Adjustments for age and sex did not change the accuracy of diagnosis (independent of MetS criteria). On the contrary, further adjustment for plasma TG decreased the accuracy of diagnosis by both lipoprotein particles regardless of diabetes or MetS definition. In brief, calculated AUC after adjustment for high TG were as following by ATPIII-MetS: 0.623(0.592, 0.653) vs. 0.644(0.616, 0.673) related to non-HDL-C and Diff-C, respectively. Using the IDF criteria, corresponding measures were 0.625(0.593, 0.657) vs. 0.616(0.586, 0.647).further details were not shown. We have also depicted the discriminatory power of LDL-C to make a comparison. Accordingly, the AUC of LDL for IDF-defined MetS was 0.608(0.588, 0.628) versus 0.617(0.597, 0.637) for ATP III-MetS.

The 5 components of MetS were analyzed separately. Accuracy of non-HDL-C for the diagnosis of each component was depicted by AUC: (high TG: 0.790), (high BP: 0.597), (hyperglycemia: 0.535), (low HDL: 0.591), (central obesity-ATP: 0.633), (central obesity-IDF: 0.644). Corresponding values for Diff-C were: 0.984, 0.611, 0.606, 0.743, 0.623 and 0.676, respectively.

**Fig. 4:**
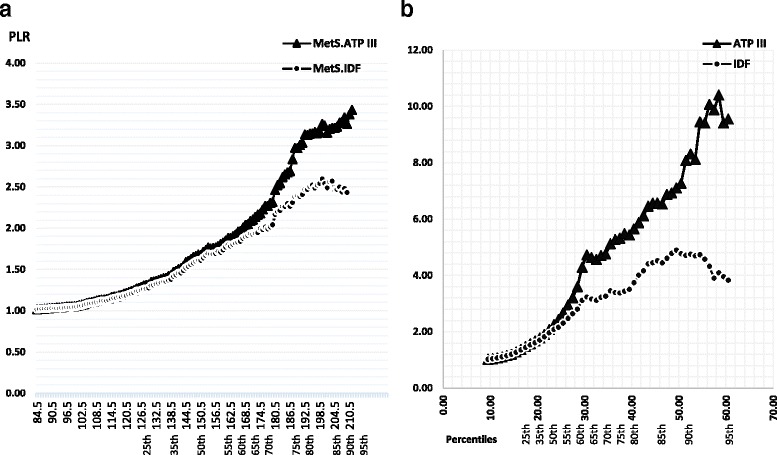
Age and sex standardized positive likelihood ratios of Non-HDL-C and Diff-C for metabolic syndrome (IDF and ATP III). Panels (**a**). and (**b**). refer to total (total Non-HDL-C) and differential fractions (Diff-C) respectively

Figure 4 shows the distribution of PLR (Positive Likelihood Ratio) according to various percentiles of non-HDL-C and Diff-C. The likelihood of metabolic syndrome increased steadily with increasing percentiles of non-HDL cholesterol and Diff-C. A threshold was determined at the 40th percentile of non-HDL-C (PLR = 1.60, at 148.5 mg/dl). For Diff-C, it was assumed at the 35th percentile (PLR = 5.42, at 20 mg/dl). Likelihood ratios for ATPIII definition were greater than for IDF, particularly at higher percentiles of non-HDL-C. It was also depicted among Diff-C percentiles. Furthermore, a similar pattern was observed in almost all categories.

**Fig. 5:**
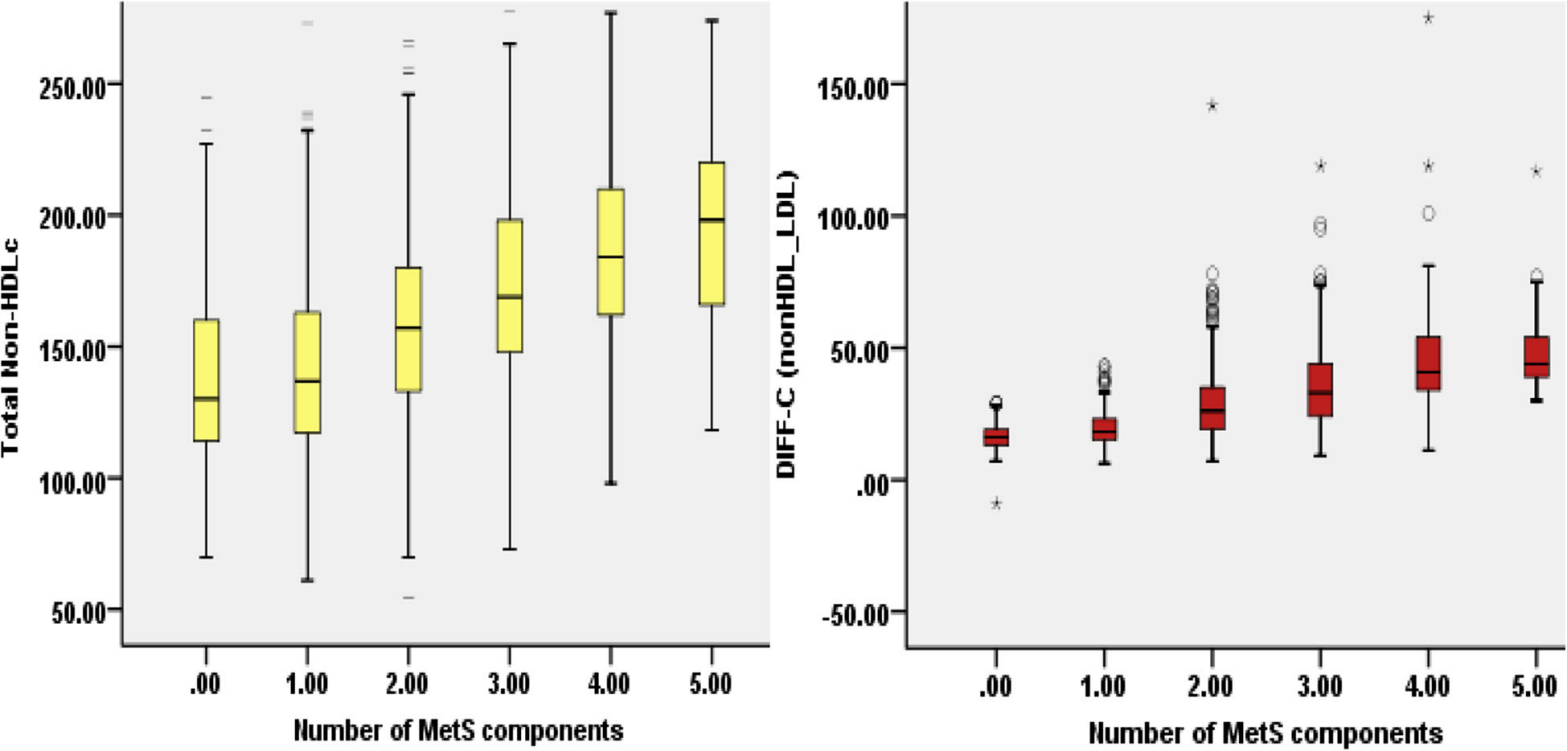
Mean levels of Non-HDL-C and Diff-C based on number of Metabolic syndrome components

Total non-HDL-C and Diff-C levels were significantly correlated (*r* = 0.54, *P* < 0.0001). Both of the surrogate measures (using means and median values) substantially increased with rising numbers of MetS components. Hence, *P*-values were <0.01 for trends of medians (Fig. 5). Mean non-HDL-C concentrations (± SE) among the categories (number of components): N0: 137.45 ± 2.90, N1: 141 ± 1.71, N2: 158.05 ± 1.48, N3: 172.43 ± 1.83, N4: 187.58 ± 3.16 and N5: 195.64 ± 4.0. Mean levels of Diff-C fraction were as following: N0: 16.05 ± 0.45, N1: 19.21 ± 0.32, N2: 28.36 ± 0.59, N3: 35.92 ± 0.77, N4: 44.20 ± 1.04 and N5: 48.31 ± 1.66. Both *P*-value for tends were <0.001.

Table 4 represents the results of multivariable logistic regression. Multiple adjustments were performed to decrease the influence of potentially confounders. Six models were designed with considerable R^2^ (pseudo-R^2^ for models 2–6 ranged from 0.52 to 55.5). As described in the table, non-HDL cholesterol was highly associated with metabolic syndrome (ORs ranged 2.7 -4.42; all *P*-values <0.0001). The relationships were confirmed for both criteria of MetS, whether traditional cut-points are applied (160 and 190 mg/dl) or not. Diff-C was also associated with MetS. Odds ratios (95% CI) for MetS in subjects with elevated Diff-C were as following: 26.29(17.71, 39.05) and 10.71(7.47, 15.35) for ATP III and IDF criteria, respectively. The Odds ratios related to Diff-C were also adjusted for age, sex, residence area, total physical activity, waist circumference, hypertension, Insulin resistance (HOMA.IR), FPG, and BMI.

**Table 4:** Odds ratios (95% CI) for MetS (ATP III, IDF) by known cutoff points of non–HDL-C

MetS definition	Models *	Non–HDL-c ≥ 160 (vs. <160)	Non–HDL-c ≥ 190 (vs <190)	Non-HDL-c quartiles¶		
				Q 2 Vs.Q 1	Q 3 vs. Q 1	Q 4 vs. Q 1
ATP III -MetS	Model 1^a^	3.89 (3.14, 4.82)	4.42 (3.48, 5.62)	2.48 (1.79, 3.44)	4.42 (3.21, 6.09)	9.68 (6.96, 13.47)
	Model 2^b^	2.96 (2.28, 3.84)	3.73 (2.79, 4.97)	1.96 (1.32, 2.93)	3.02 (2.04, 4.46)	6.60 (4.47, 9.73)
	Model 3^c^	2.77 (2.12, 3.63)	3.67 (2.68, 4.93)	1.87 (1.24, 2.83)	2.70 (1.80, 4.06)	6.12 (4.08, 9.18)
	Model 4^d^	2.75 (2.10, 3.61)	3.61 (2.67, 4.88)	1.78 (1.18, 2.70)	2.62 (1.74, 3.95)	5.87 (3.92, 8.80)
	Model 5^e^	2.77 (2.11, 3.63)	3.64 (2.68, 4.93)	1.87 (1.24, 2.84)	2.70 (1.79, 4.06)	6.13 (4.09, 9.19)
	Model 6^f^	2.76 (2.11, 3.64)	3.63 (2.68, 4.91)	1.86 (1.24, 2.84)	2.70 (1.80, 4.06)	6.11 (4.07, 9.17)
IDF -MetS	Model 1	3.82 (3.07, 4.76)	3.60 (2.83, 4.58)	2.02 (1.45, 2.81)	4.18 (3.02, 5.79)	7.64 (5.49, 10.63)
	Model 2	3.24 (2.38, 4.41)	2.82 (2.13, 3.73)	1.47 ^¥^ (0.87, 2.47)	3.18 (1.90, 5.32)	5.18 (3.17, 8.48)
	Model 3	3.14 (2.30, 4.28)	2.70 (2.32, 3.58)	1.42 ^¥^ (0.84, 2.40)	3.05 (1.82, 5.13)	4.89 (2,97, 8.05)
	Model 4	3.14 (2.30, 4.29)	2.70 (2.03, 3.59)	1.43 ^¥^ (0.85, 2.44)	3.08 (1.83, 5.19)	4.90 (3.00, 8.16)
	Model 5	3.13 (2.29, 4.28)	2.69 (2.03, 3.58)	1.42 ^¥^ (0.84, 2.41)	3.03 (1.82, 5.14)	4.89 (2.97, 8.05)
	Model 6	3.09 (2.27, 4.23)	2.68 (2.02, 3.56)	1.42 ^¥^ (0.85, 2.43)	3.04 (1.82, 5.11)	4.84 (2.94, 7.97)

*^a^In model 1, ORs were adjusted for age, sex and residential area

^b^In model 2, ORs were adjusted for covariates in model 1 plus Hypertension, total physical activity and waist circumference

^c^In model 3, ORs were adjusted for covariates in model 2 plus FBS and Insulin resistance (HOMA.IR)

^d^In model 4, ORs were adjusted for covariates in model 3 plus BMI

^e^In model 5, ORs were adjusted for covariates in model 3 plus C-reactive protein

^f^In model 6, ORs were adjusted for covariates in model 3 plus smoking (current daily smoking)

**¶**Quartiles of Non-HDL-c defined as: Q1: non-HDL-c < 132, Q2: 132–160, Q3:160–188, Q4: non-HDL-c > 188

¥ refers to a *P*-value >0.05 ﻿All other *P*-values for all of the Odds ratios were <0.0001

## Discussion

Atherosclerosis is the most common underlying cause of incident cardiovascular events particularly coronary events. As explained previously, non-HDL cholesterol provides an estimate of Apo (B) containing lipoproteins. Apo B is the core protein comprising the structure of atherogenic particles including Intermediate-density lipoprotein cholesterol (IDL-C), LDL-C, Very–low-density lipoprotein cholesterol (VLDL-C) and LP (a). Each of this non-HDL cholesterol particles has only one Apo B and About 90% of Apo B-containing particles are LDL-C, this finding shows the strong correlation between the plasma concentrations of LDL particles and number of ApoB. Apo B can modulate the transportation of cholesterol content of these particles. Entrapment of lipoproteins within the endothelial wall and further degradation initiates an inflammatory cascade leading to atherosclerotic lesions [21]. Given the pathophysiology, non-HDL-C reflects a causal relationship in addition to the role of risk assessment. Based on the utility of the metabolic syndrome in evaluation of CHD risk, the relation between non-HDL-C and MetS was examined. We demonstrated that non-HDL cholesterol (including Diff-C fraction) is strongly associated with metabolic syndrome. It was a consistent finding after multiple adjustments, in line with the majority of previous studies [22–26].

Plasma concentrations of the lipoproteins were highly related to the number of MetS components which was similar to results of previous studies [22–24]. The prevalence of MetS increased to a large degree along with rising levels of total non-HDL-C and Diff-C fractions regardless of gender type or criteria set for MetS. Hence, it was more common in the upper quartiles of non-HDL-C than the lower ones just like in high Diff-C category versus lower concentrations. We concluded that females had higher prevalence of ATP III-defined MetS than male subjects. Nevertheless, the prevalence of IDF-defined MetS in men was not significantly different than that of women. Greater proportions of IDF-defined MetS in men was confined only to the upper quartiles of non-HDL-C (compared with those in women).

It was shown that a cluster of risk factors characterized by obesity, dyslipidemia, hypertension, insulin resistance and low physical activity co-occur more commonly in diabetics (Table 1). Therefore we performed multiple adjustments for these covariates in addition to age, sex, residence area, CRP and FPG (or DM status) in logistic regression analysis. It was demonstrated that high levels of non-HDL cholesterol were associated with higher odds of developing metabolic syndrome. Particularly, individuals in the highest quartile of non-HDL-C were more likely to have MetS compared with the lowest quartile. Corresponding odds ratios were ranged as following: 6.11-9.68 and 4.84-7.64 for ATP III and IDF criteria, respectively. Furthermore, we concluded that comparative ability of Diff-C fraction is greater than total non-HDL-C to assess the presence of metabolic syndrome. Whether applying traditional or new thresholds (and goals), non-HDL cholesterol was found to have ample power in discriminating subjects with and without MetS (Table 4). “Du et al.” demonstrated that ORs (95% CI) among extreme quartiles were as following: 1.74 (1.40, 2.16) in men and 1.80 (1.47, 2.21) in women. The accuracy of non-HDL-C to identify MetS (ATP-III) was inferior to our estimates (our calculated AUCs was 0.719 compared with 0.606 and 0.602 for men and women, respectively; *P*-value <0.0001) [25]. An investigation among Korean women, also confirmed the association between non-HDL-C and MetS. Odds ratio of extreme tertiles (t3 vs. t1) for ATP-III criteria was 4.05 (1.51, 13.94), whereas using IDF criteria did not show significant results [27]. In spite of this finding, we demonstrated the associations between non-HDL-C and both definitions of MetS. However, we used the national cut-points for abdominal obesity and IDF definition leading to a significant difference.).”Kim SW” and colleagues indicated that non-HDL-C was a valuable predictor of metabolic syndrome. The accuracy measures were comparable to our results (AUC (95%CI) in males and females, respectively: 0.75(0.74, 0.76) and 0.84 (0.83, 0.85)) [22]. The same results were found in the study of “Gasevic” et al. The measures (AUCs) were greater than our estimates (0.793 and 0.818 for men and women, respectively; *P*-values <0.01) [23]. On the other hand, there are a few number of studies which have not shown any significant correlation or undermined the association of non-HDL cholesterol and MetS [28, 29].

The mechanisms linking non-HDL-C to MetS have not been completely explained though the following pathways have been proposed: low-grade inflammation, pro-coagulatory state, thrombosis, and atherosclerosis. Insulin resistance accompanied by obesity might also play a role through the development of impaired glucose tolerance and DM. Moreover, it is associated with a variety of CVD risk factors including hypertension, dyslipidemia, inflammation, endothelial dysfunction and chronic kidney disease [30]. However, the association of non-HDL-C and MetS remained significant and robust despite controlling the effects of such determinants (models 1–6). It may denote the existence of additional mechanisms for the residual risk of having MetS. Nevertheless, adjustment for elevated TG diminished the ORs substantially (in almost all categories; data not shown). Given this point, Hypertriglyceridemia is thought to be an essential confounder augmenting the association of non-HDL-C and MetS.

Greater odds of having MetS computed for elevated Diff-C may address the lack of a robust relationship between LDL-C and MetS. Furthermore, the connection of atherogenic lipoprotein phenotypes (non-HDL-C complex) and obesity is more powerful than that of LDL-C [31, 32]. Also, the accuracy of LDL-C for the diagnosis of metabolic syndrome was notably lower as compared with non-HDL cholesterol. This finding was independent of DM and MetS criteria (data not shown). It is consistent with the evidence indicating the superiority of non-HDL-C (and apoB) to LDL-C [6].

Subsidiary analyses among the 5 components of MetS shows the following: both total non-HDL-C and Diff-C levels are most strongly associated with high TG concentration (the maximal AUC) while the smallest AUC pertains to hyperglycemia. Additionally, performing adjustment for elevated TG in ROC analysis resulted in lowering the area under the curve. Hence, the accuracy of non-HDL-C and Diff-C fractions to distinguish between cases with and without metabolic syndrome notably decreased. In the same way, further adjustment for high TG (>150) led to considerable reduction in ORs of having MetS among non-HDL-C subgroups (regardless of MetS criteria). Contribution of non-HDL-C to hypertriglyceridemia as evidenced in this study, lies in line with previous reports [22, 33]. The association of atherogenic lipid profile and high plasma triglyceride may serve as a major culprit in progression of atherosclerosis. TG-rich particles especially VLDL-C synthesized by the liver (during fasting periods) and other apoB-containing fractions comprise the majority of non-HDL-C complex. Fasting (as considered in this study) limits the intestinal origin of plasma TG (carried in chylomicrons) [34]. Also, correlation of non-HDL-C and high TG can be described through the following pathways: First, co-occurrence of hypertriglyceridemia, low HDL-C and high levels of small dense-LDL particles known as the atherogenic lipoprotein phenotype [35]. Second, the potential modulating role of both conditions in adiposity [36]. Third, enhanced hepatic synthesis of apoB and VLDL-C might be triggered by insulin resistance. Furthermore, glucose intolerance and DM may deal with this association. Increased influx of peripheral free fatty acids into the liver, induced production of VLDL, diminished performance of lipoprotein lipase and increased transportation of particles have been suggested as underlying mechanisms [21, 37]. However, the remained controversy is about the benefits and utility of treatments in hypertriglyceridemia to prevent CHD [38].

Concomitant increase of plasma TG and apoB level accounts for the mainstay of dyslipoproteinemia serving as a major determinant of both DM and MetS. Furthermore, it is the most common phenotype observed in premature coronary heart disease [39]. ApoB index reflects the number atherogenic particles, whereas the non-HDL-C indicates the mass of cholesterol content. However, the great correlation of apoB and non-HDL-C suggests to use both parameters in clinical practice [7]. The Adult Treatment Panel IV (ATP IV) guidelines from the National Cholesterol Education Program (NCEP), does not clearly measure the power of apoB against non–HDL-C. Collective results support the valuable role of both non-HDL-C and apo-B in primary and secondary risk assessments regardless of serum triglyceride concentrations and content of the meals. Superior benefits of non-HDL-C as compared with apo-B are related to simple calculation, more achievable practical cut-offs and well-defined treatment goals [40].

Major Limitation of the present study refers to the cross-sectional design which is not able to determine the causality of associations. In addition, the clinical importance of using lipoprotein fractions (Non-HDL-C and Diff-C) could be revealed by including more definite end points (such as coronary artery disease or mortality instead of metabolic syndrome which is an intermediate indicator of cardiovascular disease).

## Conclusion

In conclusion, components of atherogenic lipid profile can help to yield the clinical goals including prevention and early diagnosis of CHD events. With regard to racial and genetic heterogeneities between and within populations, specific cut-points of lipids might be valuable. The optimal cut-points of non-HDL-C and Diff-C that we have proposed (for both IDF- and ATP III-defined MetS) are about 153 and 30, respectively. Determined points for non-HDL-C are greater in diabetics than nondiabetics. The accuracy of both surrogate measures to identify MetS are greater in nondiabetic subjects. It is worth noting that metabolic syndrome was primarily more prevalent in diabetics. Meanwhile higher concentrations of non-HDL cholesterol fractions were found in diabetics. Specific thresholds in men and women were not significantly different (with respect to their similar non-HDLC levels). Therefore, cut-points determined for general population and DM subgroups are acceptable in both genders. ATPIII-defined MetS was predicted more accurately than IDF-defined MetS and the discriminatory power of Diff-C was higher than that of non-HDL-C. Along with increasing percentiles of both measures, positive likelihood ratios of MetS increased substantially while the risk gradients were greater for ATP III criteria.

## Abbreviations

AUC: Area under the curve
BMI: Body mass index
CHD: Coronary heart disease
CVD: Cardiovascular disease
Diff-C: Differential fraction cholesterol (difference of total non-HDL-C and LDL-C)
IDL-C: Intermediate-density lipoprotein cholesterol
LDL-C: Low-density lipoprotein cholesterol
Ln-CRP: Natural logarithm of C-reactive protein
MetS: Metabolic syndrome
Non–HDL-C: Non–high-density lipoprotein cholesterol
ROC: Receiver operating curve
VLDL-C: Very–low-density lipoprotein cholesterol
WC: Waist circumference
